# Do intrapersonal factors mediate the association of social support with physical activity in young women living in socioeconomically disadvantaged neighbourhoods? A longitudinal mediation analysis

**DOI:** 10.1371/journal.pone.0173231

**Published:** 2017-03-16

**Authors:** Anouk Middelweerd, Saskia J. te Velde, Gavin Abbott, Anna Timperio, Johannes Brug, Kylie Ball

**Affiliations:** 1 Department of Epidemiology & Biostatistics and the EMGO+ Institute for Health and Care Research, VU University Medical Center, Amsterdam, the Netherlands; 2 Institute for Physical Activity and Nutrition, School of Exercise and Nutrition Sciences, Deakin University, Geelong Victoria, Australia; 3 Amsterdam School of Communication Research, University of Amsterdam, Amsterdam, the Netherlands; Leibniz Institute for Prvention Research and Epidemiology BIPS, GERMANY

## Abstract

**Background:**

Levels of physical activity (PA) decrease when transitioning from adolescence into young adulthood. Evidence suggests that social support and intrapersonal factors (self-efficacy, outcome expectations, PA enjoyment) are associated with PA. The aim of the present study was to explore whether cross-sectional and longitudinal associations of social support from family and friends with leisure-time PA (LTPA) among young women living in disadvantaged areas were mediated by intrapersonal factors (PA enjoyment, outcome expectations, self-efficacy).

**Methods:**

Survey data were collected from 18–30 year-old women living in disadvantaged suburbs of Victoria, Australia as part of the READI study in 2007–2008 (T0, N = 1197), with follow-up data collected in 2010–2011 (T1, N = 357) and 2012–2013 (T2, N = 271). A series of single-mediator models were tested using baseline (T0) and longitudinal data from all three time points with residual change scores for changes between measurements.

**Results:**

Cross-sectional analyses showed that social support was associated with LTPA both directly and indirectly, mediated by intrapersonal factors. Each intrapersonal factor explained between 5.9–37.5% of the associations. None of the intrapersonal factors were significant mediators in the longitudinal analyses.

**Conclusions:**

Results from the cross-sectional analyses suggest that the associations of social support from family and from friends with LTPA are mediated by intrapersonal factors (PA enjoyment, outcome expectations and self-efficacy). However, longitudinal analyses did not confirm these findings.

## Introduction

Although the positive health effects of physical activity (PA) are well known [1–4], only approximately 30% of the Australian adult population meet the recommended guidelines of at least 30 minutes of moderate-intensity PA per day. [5] Only 48.7% of young Australian women aged 18–24 years meet the recommended PA guidelines and the percentages tend to decline substantially in older age groups. [5, 6] Previous research has shown that PA levels decrease during the transition from adolescence into adulthood. [6, 7] Life events that occur in young adulthood, such as leaving school and home, getting married or having children, may be associated with disruption in PA during this life stage [7], especially for women. [8, 9] Furthermore, women living in the most socioeconomically disadvantaged areas already are less physically active than those from more advantaged neighbourhoods, and thus are even more at risk of adverse health outcomes resulting from age-related declines in PA. [10]

In order to minimise reductions in PA in young adults living in disadvantaged neighbourhoods, we need to understand the determinants of PA in this population group.

Various social-cognitive and ecological-based models have been published which aim to predict and understand health behaviours.[11] These models and theories describe a range of potential behavioural determinants, e.g. environmental factors such as availability and socio-cognitive factors such as self-efficacy and social support. Often used theories and models are the Social Cognitive Theory (SCT)[12–14] and socio-ecological models [15]. These models postulate that the social environment influences health behaviours, such as PA, both directly and indirectly (i.e. through self-efficacy). That the social environment is indeed an important determinant of PA has been confirmed in observational research.[16–18]Moreover, self-efficacy, or similar constructs, is a key construct within various social cognitive theories and has consistently positively been associated with PA. [18, 19] Outcome expectations, another key concept in the SCT, represent one’s beliefs about the consequences and perceived benefits of one’s behavior such as participating in PA and has consistently been associated with PA. [20]

In addition, expected enjoyment from physical activities, which can be seen as an indicator of intrinsic motivation, has been positively associated with PA. [21] Overall, cognitive factors (e.g., self-efficacy, outcome expectations (i.e., perceived health benefits) and expected PA enjoyment) and social support have been found to be positively associated with PA levels. [11, 18, 22, 23] However, the underlying mechanisms and inter-relationships by which these factors might influence PA are largely unknown. A better insight in these inter-relationships would further improve theoretical models and our understanding of PA and other health behaviours. [22, 24, 25] Recent studies indicate that cognitive factors such as self-efficacy mediate the association of social support and PA. [25–27]. This means that social environmental factors, such as social support, would positively influence cognitive factors such as self-efficacy, which in turn positively influences PA. However, these results are derived from cross-sectional mediation studies and should be interpreted with caution due to their inability to identify temporal or causal effects and pathways. [28] Consequently, there is a need for longitudinal and experimental approaches aiming to explore the mechanisms underlying relationships between social factors and PA. As there are various important potential mediators described in models, theories and the literature, the current study examines a subset of these potential mediators that were available in the cohort study. The current study aimed to examine the associations between social support from family and friends with leisure time PA (LTPA) in young Australian women (aged 18–30 years) living in disadvantaged neighbourhoods and to explore whether these associations were mediated by intrapersonal factors (i.e. PA enjoyment, outcome expectations, self-efficacy) using cross-sectional and longitudinal data. We hypothesized that the association of social support and LTPA would be mediated by cognitive factors. That is, a supportive network of family and friends can facilitate LTPA, because social support may also promote PA enjoyment, outcome expectations, and self-efficacy to be physically active.

## Methods

### Procedure and participants

This study used data from the Resilience for Eating and Activity Despite Inequality (READI) cohort. The READI study collected self-reported data using mailed surveys in 2007–08 (baseline = T0), 2010–2011 (3 year follow-up = T1) and 2012–2013 (5 year follow-up = T2). [29] The study was developed to examine socioeconomic variations in PA and diet in Australian women aged 18–46 years living in disadvantaged neighbourhoods. [29] Participants were recruited from 80 socioeconomically disadvantaged neighbourhoods (40 urban and 40 rural) via a stratified random sampling approach using the electoral roll in Victoria Australia. Detailed information about the READI cohort is provided in the cohort profile. [29] The present study will solely report results from women aged 18–30 years at T0, and uses T0 (N = 1197), T1 (N = 348) T2 (N = 267) data.

The study protocol was approved by the Deakin University Human Research Ethics Committee, the Victorian Department of Education and the Catholic Education Office, and informed consent was obtained from each participant before the study started.

### Measurement

Analyses are based on survey data of LTPA, social support from family and from friends, PA enjoyment, self-efficacy, outcome expectations and demographic characteristics. Appendix 1 shows a summary of the social support and intrapersonal measures and their internal reliability and test-retest reliability.

#### Outcome variable

Physical activity was assessed at all three time points using the International Physical Activity Questionnaire Long version (IPAQ-L), which has acceptable measurement properties. [30] LTPA, the outcome variable, was assessed with six items of the IPAQ-L where participants reported time spent in walking, moderate- and vigorous-intensity PA in their leisure time during the past 7 days. LTPA was a sum-score of minutes per week of walking, or in moderate- and vigorous-intensity PA during leisure time.

#### Predictor variable

Perceived social support for PA from friends was assessed with two items using a 5-point Likert scale (1 Never– 5 Very often). [31] Whereas, social support from family was assessed using two items and a 6-point Likert scale (1 Never– 5 Very often. 6 Not Applicable).[31] Sum scores were computed for social support from family and for social support from friends for each time point (range = 2–10). For social support from family, ‘ Not Applicable’ was treated as missing and therefore did not contribute to the summed score.

#### Mediator variables

PA Enjoyment was assessed with six items using a 7-point scale assessing how the participants feel about PA at the moment (e.g. 1 I hate it– 7 I enjoy it; 1 I feel bored– 7 I feel interested). [32] A sum score (range = 6–42) was computed for each time point (Cronbach’s α = 0.93–0.95).

Physical activity outcome expectations were assessed with six items using a 4-point Likert scale (1 No reason at all– 4 A very important reason). The items captured how important health, appearance, weight, feeling fit, relaxation and stress relief are for being physically active. [25] A sum score (range = 6–24) was computed for each time point (Cronbach’s α = 0.75–0.79).

Self-efficacy for PA was assessed with five items using a 5-point Likert scale (1 Not at all confident– 5 extremely confident) assessing how confident the participants are that they could do PA in different situations (e.g. “…even when I am tired”). [33] A sum score (range = 5–25) was computed for each time point (Cronbach’s α = 0.80–0.86).

#### Demographics

Information about age, education level (low: <Year 12; medium: Year 12/trade/certificate; High: university/postgraduate), marital status (married/living as married; separated/ divorced/widowed; never been married), and children living at home (yes/no) were collected in the survey.

### Analyses

For the three time points, means and standard deviations or proportions were calculated for all variables. Repeated measures ANOVA were conducted to examine whether LTPA, social support from family and friends, PA enjoyment, outcome expectation, and self-efficacy changed over time (T0-T1-T2).

**Fig 1:**
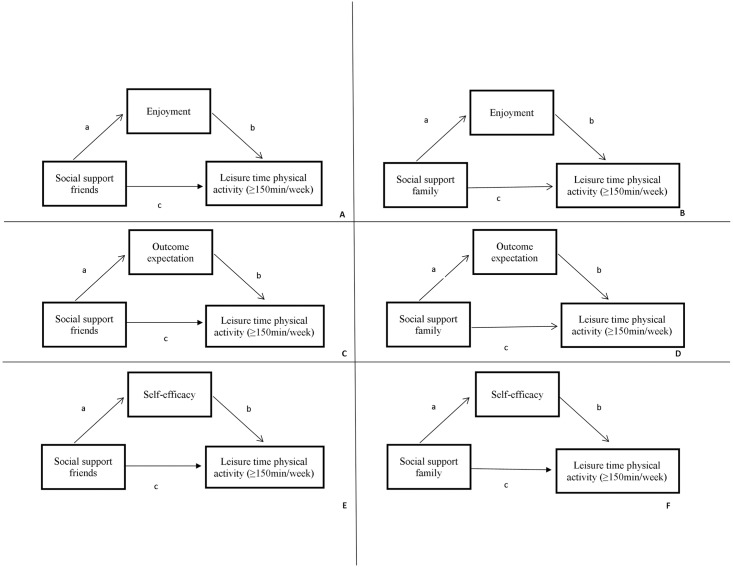
Conceptual mediation model for the cross-sectional analyses. **A-F** Conceptual model of the direct and indirect association of social support (i.e. from friends/colleagues and from family) personal determinants (PA enjoyment, self-efficacy and outcome expectations) and leisure-time physical activity (≥150minutes/week) for the cross-sectional analyses.

**Fig 2:**
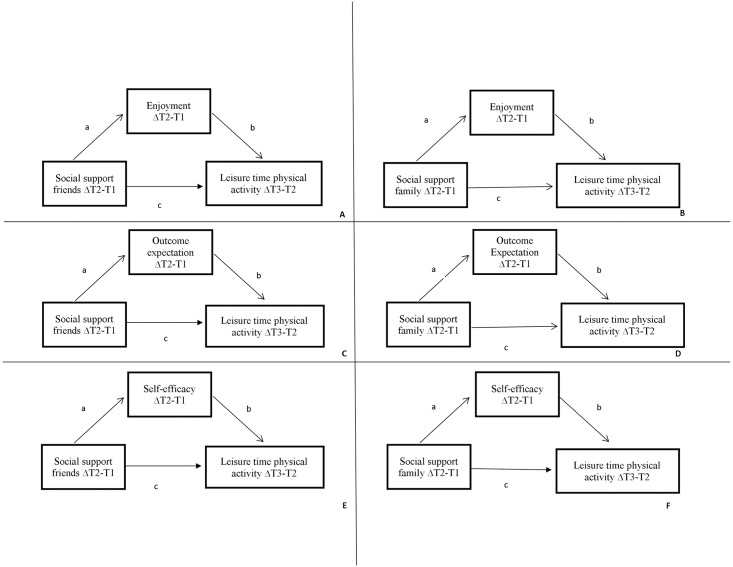
Conceptual model for the longitudinal analyses using residual change scores. **A-F** Conceptual models of the direct and indirect association of changes in social support from friends and from colleagues and family, changes in personal determinants (PA enjoyment of physical activity, self-efficacy for physical activity and outcome expectations and changes in leisure-time physical activity.

Analyses were conducted to examine associations between social support from family and friends and LTPA and whether these associations were mediated by three intrapersonal factors (PA enjoyment, outcome expectation, self-efficacy). Single mediator analyses were separately conducted for two exposures: social support from family and from friends; thus in total six mediation models were tested to explore whether the associations for social support and LTPA were mediated by intrapersonal factors (Fig 1). It is commonly considered that mediation is present when a potential mediator is significantly associated with the predictor variable and with the outcome variable while adjusting for the predictor. [34] Therefore, a series of regression analyses were conducted using a four step process: 1) the total association of social support with LTPA was estimated (path *c*, Figs 1 and 2); 2) the independent association of social support with LTPA adjusted for the potential mediators were estimated (path *c’* and *b*, Figs 1 and 2); 3) the association of social support with potential mediators was estimated (path *a*, Figs 1 and 2); and 4) the mediated effect was calculated as the product of the *a* and *b* paths, and bias-corrected bootstrapping was used to produce 95% confidence intervals of the mediated effect. [35] The proportion mediated effect was calculated as the product of *a* and *b* paths divided by *c* path.

In the first step, a cross-sectional analyses were conducted using the baseline data (T0) from 1197 participants. LTPA (at T0) was dichotomized at 150 minutes per week due to the zero-inflated distribution. The associations of social support with each potential mediator were estimated (path a, Fig 1) with linear regression models and the total and independent associations (path b, c and c’) were estimated with logistic regression models. In order to estimate the mediated effect the regression coefficient of the *c* path needed to be standardized. [36]

By definition, mediation implies change over time, which cannot be captured in cross-sectional designs. Cross-sectional data analyses can therefore lead to over- or under-estimated effects. [28, 37] To overcome this, longitudinal mediation models were used that accounted for changes over time in predictors, mediators and outcomes and their associations. To optimally use the available data, a model as depicted in Fig 2 was used. Change scores—using a continuous scale—were calculated as residual changes scores for changes between two measurements in minutes per week (i.e., XΔT0-1, MΔT0-1, YΔT1-2) as proposed by Twisk and Proper. [38] These longitudinal models used data from 271 women who completed surveys at all three time points. Changes in social support between T0-T1 were hypothesized to predict changes in LTPA between T1-T2 with changes in intrapersonal factors between T0-T1 as possible mediators (Fig 2).

Single mediation analyses were conducted using both cross-sectional and longitudinal data. As the sample size in the longitudinal data set was too limited to conduct multiple mediation analyses, for consistency and comparability we did not perform multiple mediation models with the cross-sectional data either, allowing the results from the cross-sectional and longitudinal data to be comparable. All analyses were adjusted for women’s level of education, whether they had any children living at home, and area of residence (urban or rural); robust standard errors were used to account for potential clustering within the 80 neighbourhoods. Analyses were conducted in 2015 using Stata 12 (StataCorp, TX).

#### Non-response analyses

Because of the high attrition rate, a response analyses was conducted to compare baseline characteristics between responders (N = 271) and non-responders (N = 926). Non-responders were those who either dropped out at T1 (N = 840) or at T2 (N = 86). No significant differences between groups were found, except for age and region of residence. The non-responders were on average younger (24.0 vs 24.7 years, p = 0.005) and a higher percentage lived in urban areas (58.8% vs 48.0%, *p* = 0.003). Results of the non-response analyses are presented in Appendix 2.

## Results

Women’s mean age at baseline was 24.2 years, and about 60% of the sample had an intermediate level of education (i.e., completed high school, a trade certificate or diploma), were not married, and had no children living with them. Table 1 provides additional participant characteristics.

**Table 1:** Participant’s characteristics for all three time points (T0-2).

	T0 (2007–08)	T1 (2010–11)	T2 (2012–13)
**Number of participants [N]**	1197	357	271
**Age [years]**	24.2 (3.4)	27.2 (3.4)	29.2 (3.4)
**BMI [kg/m^2^]**	24.5 (5.8)	25.4 (6.0)	25.7 (6.4)
**Education [%]**			
**High**^1^	27	38	47
**Medium**^2^	63	60	51
**Low**^3^	9	2	1
**Marital status [%]**			
** Married/living as married**	38	51	57
** Separated/divorced/widowed**	3	3	3
** Never married**	59	46	40
**Children living with you [Yes; %]**	33	39	40

*Note*. T0 = baseline, T1 = 3 year follow-up, T2 = 5 year follow-up

^1^ High education level: university/postgraduate

^2^ Medium education level: Year 12/trade/certificate

^3^ Low education level: <Year 12

There were no significant differences across the three time points for LTPA, social support from family and friends or intrapersonal factors as indicated by the non-significant F-values (Table 2).

**Table 2:** Descriptive statistics (number of participants (N), percentages (%), means and Standard Deviations (SD) and) for the three time points (T0-2), changes between two time points (T1-T0, T2-T1) and the time-effects.

Measurement	Number of participants (N)	Leisure-Time Physical activity [min/week]	Leisure-Time Physical activity (> = 150 minutes/week)	Social Support Family	Social Support Friends	Enjoyment	Outcome Expectancy	Self-Efficacy
Time point	N	Mean (SD)	N (%)	Mean (SD)	Mean (SD)	Mean (SD)	Mean (SD)	Mean (SD)
T0	1197	228.0 (250.6)	567 (48.6%)	6.1 (2.2)	4.8 (2.2)	31.3 (7.3)	18.4 (3.7)	13.5 (4.3)
T1	348	231.5 (279.0)	173 (49.4%)	6.1(2.1)	5.0 (2.3)	31.8 (7.4)	18.5 (3.4)	13.4 (4.4)
T2	267	201.8 (221.9)	129 (48.3%)	6.0 (2.3)	4.9 (2.3)	31.8 (8.3)	18.8 (3.4)	13.6 (4.9)
ΔTime points^1^	N	Mean (Range)	N (%)	Mean(Range)	Mean (Range)	Mean (Range)	Mean (Range)	Mean (Range)
T_1-0_	348	-1.2 (-1020; 1560)	NA^2^	-0.0 (-5;8)	0.1 (-8;8)	0.2 (-28;23)	0.2 (-12; 11)	-0.1 (-15; 14)
T_2-1_	267	-25.5 (-1050;1320)	NA^2^	-0.0 (-6;7)	-0.2 (-7;8)	-0.2 (-27;19)	0.3 (-12;9)	0.0 (-14;18)
Time-effects	N	F (*p-value)* ^3^	F (*p-value)* ^3^	F (*p-value)* ^3^	F (*p-value)* ^3^	F (*p-value)* ^3^	F (*p-value)* ^3^	F (*p-value)* ^3^
	235	1.43 (*p* = 0.24)	NA^2^	0.3 (*p* = 0.74)	0.94 (*p* = 0.39)	0.72 (*p* = 0.49)	1.94 (*p* = 0.15)	0.06 (*p* = 0.94)

*Note*. T0 = baseline, T1 = 3 year follow-up, T2 = 5 year follow-up

^1^ Changes in Leisure-time physical activity, social support from family and from friends, PA enjoyment, outcome expectations and self-efficacy between measurement at T0 and T1 (T1-T0) and between T1 and T2 (T2-T1), respectively.

^2^ Not applicable (NA) for difference between time points

^3^ F-value calculated with the repeated measures ANOVA with *df*_*time*_ = 2, *df*_*residuals*_ = 468 and the *p*-value

### Association of social support variables with leisure-time physical activity (c-path)

Cross-sectional logistic regression analyses showed that social support from family and friends were both significantly positively associated with participation in ≥150 minutes per week of LTPA (Table 3). Longitudinal analyses showed no significant associations of changes in social support from family and friends from baseline (T0) to 3 year follow-up (T1), with changes in LTPA from 3 year follow-up (T1) to 5 year follow-up (T2) (Table 4).

**Table 3:** Results from single mediation models (Odds Rations (OR) and regression coefficients (B), with 95% confidence intervals (95%CI)) examining potential intrapersonal mediators of leisure time physical activity (> = 150minutes/week)^1^ for cross-sectional data at baseline.

Independent variable	Potential mediator	c-path OR (95%CI)^2^^,^^8^	a-path B (95%CI)^3^^,^^8^	b-path OR (95%CI)^4^^,^^8^	c'-path OR (95%CI)^5^^,^^8^	Mediated effect a*b (95%CI)^6^	Proportion mediated (a*b)/c ^7^
**Family social support**	Enjoyment	**1.18 (1.11; 1.25)**	**0.64 (0.43; 0.86)**	**1.11 (1.09; 1.13)**	**1.13 (1.06; 1.20)**	**0.07 (0.04;0.09)**	37.5%
	Outcome Expectations	**1.18 (1.11; 1.24)**	**0.14 (0.07; 0.23)**	**1.09 (1.05; 1.13)**	**1.16 (1.10; 1,23)**	**0.01 (0.00;0.02))**	7.8%
	Self-Efficacy	**1.18 (1.11; 1.25)**	**0.32 (0.20; 0.43)**	**1.22 (1.19; 1.26)**	**1.13 (1.06; 1.21)**	**0.06 (0.04;0.09)**	35.9%
**Friend social support**	Enjoyment	**1.25 (1.18; 1.33)**	**0.66 (0.51; 0.82)**	**1.11 (1.08; 1.13)**	**1.20 (1.12; 1.28)**	**0.07 (0.05;0.09)**	28.3%
	Outcome Expectations	**1.25 (1.18; 1.32)**	**0.15 (0.06; 0.24)**	**1.09 (1.05; 1.13)**	**1.24 (1.17; 1.32)**	**0.01 (0.00;0.02)**	5.9%
	Self-Efficacy	**1.25 (1.18; 1.33)**	**0.40 (0.30; 0.50)**	**1.22 (1.18; 1.25)**	**1.20 (1.13; 1.28)**	**0.08 (0.05;0.10)**	31.5%

*Note*. Values represented in bold are significant associations

^1^ The dependent variable leisure time physical activity was dichotomized at 150 minutes activity per week

^2^ c-path (total effect): is the association of social support from family or friends and leisure time physical activity analysed with a logistic regression analyses

^3^ a-path: the association between social support from family or friends and mediator analysed with a linear regression analyses

^4^ b-path: the association of mediator and leisure time physical activity analysed with a logistic regression analyses

^5^ c’-path (direct effect) is the association of social support from family or friends and leisure time physical activity adjusted for the mediator. This association is analysed with a logistic regression analyses.

^6^ Mediated effect (a*b) is the indirect effect of the independent variable on the outcome variable through the potential mediator.

^7^ Proportion mediated effect (a*b/c) using the adjusted *c* coefficient

^8^ All analyses are models adjusted for educational level (high, medium, low), living with children (yes, no), living area (rural vs urban) and robust standard errors were used to account for potential clustering within the neighbourhoods

**Table 4:** Results from longitudinal single mediation models (regression coefficients (B) and 95% confidence intervals (95%CI)) examining potential intrapersonal mediators of leisure time physical activity using residual change scores.

Independent variable	Potential mediator	c-path B (95%CI)^1^^,^^7^	a-path B (95%CI)^2^^,^^7^	b-path B (95%CI)^3^^,^^7^	c'-path B (95%CI)^4^^,^^7^	Mediated effect a*b (95%CI)^5^	Proportion mediated (a*b)/c ^6^
**Family social support**	Enjoyment	-4.06 (-16.68; 8.57)	0.28 (-.05; 0.60)	-3.00 (-1.25; 7.24)	-4.88 (-17.11; 7.35)	0.82 (-0.86; 2.51)	-
	Outcome Expectations	-3.90 (-16.00; 8.20)	**0.21 (0.04; 0.37)**	6.39 (-4.01; 16.79)	-5.21 (-17.98; 7.55)	1.31 (-1.11; 3.73)	-
	Self-Efficacy	-3.29 (-15.50; 8.92)	0.15 (-0.08; 0.39)	0.39 (-9.42; 10.20)	-3.35 (-15.36; 8.67)	0.06 (-1.82; 1.94)	-
**Friend social support**	Enjoyment	-7.91 (-21.98; 6.16)	0.28 (-0.01; 0.57)	3.33 (-0.93; 7.59)	-8.83 (-22.27; 4.61)	0.92 (-0.74; 2.58)	-
	Outcome Expectations	-8.42 (-22.32; 5.47)	0.09 (-0.10; 0.28)	6.61 (-3.28; 16.49)	-9.03 (-23.346; 5.19)	0.60 (-1.06; 2.26)	-
	Self-Efficacy	-8.02 (-22.11; 6.07)	**0.30 (0.08; 0.52)**	1.16 (-8.54; 10.86)	-8.37 (-21.95; 5.21)	0.35 (-2.60; 3.30)	-

Note. Values represented in bold are significant associations

^1^ c-path (total effect): is the association of changes in social support from family or friends between baseline and 3 year follow-up and changes in leisure time physical activity between 3 year and 5 year follow-up analysed with a linear regression analyses using residual change scores

^2^ a-path: the association between changes in social support from family or friends between baseline and 3 year follow-up and changes in the mediator between baseline and 3 year follow-up analysed with a linear regression analyses using residual change scores

^3^ b-path: the association of changes in the mediator between baseline and 3 year follow-up and changes in leisure time physical activity between 3 year and 5 year follow-up analysed with a linear regression analyses using residual change scores

^4^ c’-path (direct effect) is the association of changes in social support from family or friends between baseline and 3 year follow-up and changes in leisure time physical activity between t 3 year and 5 year follow-up adjusted for changes in the mediator between baseline and 3 year follow-up. This association analysed with a linear regression analyses using residual change scores

^5^ Mediated effect (a*b) is the indirect effect of the independent variable on the outcome variable through the potential mediator

^6^ Proportion mediated effect (a*b/c)

^7^ All analyses are models adjusted for educational level (high, medium, low), living with children (yes, no), living area (rural vs urban) and robust standard errors were used to account for potential clustering within the neighbourhoods

### Association of social support variables with intrapersonal factors (a-path)

Cross-sectional linear regression analyses showed that social support from family and friends were both significantly positively associated with PA enjoyment, outcome expectancy and self-efficacy for PA at baseline (Table 3). Longitudinal analyses showed that changes in social support from family between T0 and T1 were positively significantly associated with changes in outcome expectations between T0 and T1 (B = 0.21, 95%CI = 0.05–0.37) (Table 4). Changes in social support from friends from T0-T1 were positively significantly associated with changes in self-efficacy from T0-T1 (B = 0.29, 95%CI = 0.08–0.51) (Table 4).

### Association of intrapersonal factors with leisure-time physical activity (b-path)

Cross-sectionally, all hypothesized mediators (PA enjoyment, outcome expectations, self-efficacy) were significantly positively associated with LTPA adjusted for social support from friends. Furthermore, all hypothesized mediators (PA enjoyment, outcome expectations, self-efficacy) were significantly positively associated with LTPA adjusted for social support from family (Table 3) as well. Longitudinal analyses showed that none of the changes between T0 and T1 in the hypothesized mediators (PA enjoyment, outcome expectations, self-efficacy) were significantly associated with changes in LTPA from T1-T2 adjusted for either changes in social support from family or friends from T0-T1 (Table 4).

### Mediating effects of intrapersonal factors

Results from cross-sectional analyses showed that all hypothesized mediators (PA enjoyment, outcome expectations, self-efficacy) had significant roles as mediators, with each intrapersonal factor mediating between 5.9–37.5% of the total associations. Results from the longitudinal analyses showed that none of the intrapersonal factors (PA enjoyment, outcome expectations, self-efficacy) had significant roles as mediators (Table 4).

## Discussion

The current study aimed to examine the associations of social factors (social support from family and from friends) and LTPA in young Australian women (aged 18–30 years) living in disadvantaged neighbourhoods and to explore whether these associations were mediated by PA enjoyment, outcome expectations and self-efficacy, using cross-sectional and longitudinal data. Our hypothesis that the association of social support and LTPA was mediated by these intrapersonal factors was partly supported. Cross-sectional analyses indicated that all intrapersonal factors (PA enjoyment, outcome expectations, self-efficacy) were significant mediators in the associations of social support from family and from friends with LTPA. However, longitudinal analyses did not support the hypothesis that changes in social support from family or from friends and subsequent changes in LTPA, were mediated by changes in PA enjoyment, outcome expectations, or self-efficacy.

Cross-sectional analyses indicated that self-efficacy was a mediator of the associations of social support from family and from friends and LTPA, which is in line with previous studies. [22, 26, 39] A prior study in undergraduate students, for example, demonstrated that the association of social support from friends with PA was fully mediated by self-efficacy, using baseline and three-week follow-up data. [22] Furthermore, a study among adults aged 18–92 supported the hypotheses that the association of social support with PA was mediated by self-efficacy. [39] Additionally, Aghdam et al[26] reported that social support directly and indirectly through self-efficacy affected moderate and vigorous PA in Iranian women aged 36.8 years on average. Thus, results from previous studies and from this study indicate that social support from family or from friends—such as encouragement or co-participation—are associated with an increase in PA partly because levels of self-efficacy increase as well. A plausible explanation may be that family or friends provide verbal encouragement, persuasion and also provide vicarious experiences. Both verbal persuasion and vicarious experience are seen as social influences and are known sources of self-efficacy. [19]

Cross-sectional analyses indicated that enjoyment was a mediator of the associations of social support from family and from friends and LTPA, To our knowledge, ours is the first study that examined the mediating effect of PA enjoyment in the association of social support from family or from friends and LTPA. However, several studies examined the association between enjoyment and PA and between social support and enjoyment for PA. Motl et al[40] demonstrated that PA enjoyment was directly associated with PA and indirectly associated with PA through self-efficacy in patients with multiple sclerosis indicating the associations might even be more complex. Moreover, previous qualitative studies indicated that PA was influenced by social support and PA enjoyment. [41] Furthermore, Duin et al[42] reported that social support was an important factor for participating in physical activities, and that social events—including being physically active with others—were reasons to engage in physical activities even if participants did not like the sports. Consequently, having family or friends who co-participate in physical activities might increase levels of PA, because levels of enjoyment increase due to the fact that exercising together is a social event, or because of the social interaction. Furthermore, a supportive and encouraging network can be perceived as a form of positive reinforcement [43], that could increase PA enjoyment and consequently increase PA.

Lastly, we hypothesized that a supportive network of family and friends can facilitate LTPA because social support may positively influence the women’s outcome expectations of PA. Again, this hypothesis was only supported by the results from the cross-sectional analyses. Such mediation has not been studied before, but previous research examining the association of outcome expectation and PA and the placement of outcome expectation in health models showed mixed results. [44] For example, Anderson et al[39] showed that outcome expectations partly mediated the association of self-efficacy and PA in adults. Furthermore, Ayotte et al[45] reported that social support was directly and indirectly associated with outcome expectations through self-efficacy and social support was directly and indirectly associated with PA through self-efficacy. These results indicate that more complex mediation models may be in place and this needs further examination.

None of the cross-sectional evidence for mediation was supported by the longitudinal analyses. The first reason for this may be that the cross-sectional analyses merely indicate associations, but not true mediation. However, the longitudinal analyses also have their limitations, and these are not per definition superior to the cross-sectional analyses in the present study. One issue is that we used change scores for the longitudinal analyses, and that the changes over time were not substantial enough to identify mediated pathways. Additionally, it is not clear what the ideal time laps is for assessing change in determinants and potential mediators –factors like enjoyment, self-efficacy, outcome expectations and perceived social support may have changed repeatedly as well as back and forth between baseline and first follow-up, and this may have obscured predictive and mediation effects over the longer period of time as used for the analyses in the present study. To further investigate the mediators hypothesized in the present study and to strengthen the evidence for causality, intervention studies that induce changes in self-efficacy, PA enjoyment and outcome expectations are needed, with repeated and frequent assessments of both mediators and outcomes. Mediation analyses aim to examine causal pathways and thus mediation models assume specific temporal ordering of the variables. In cross-sectional analyses the presumed time ordering among the variables is based on theory and previous findings; all variables are measured at the same moment and therefore cross-sectional analyses can only estimate the associations based on differences between individuals. Longitudinal analyses have the ability to identify temporal effects and examine the changes within individuals in addition to differences between individuals. [36, 46] Furthermore, previous studies indicate that the associations may be more complex than those tested here. Therefore, studies using alternative modelling approaches such as structural equation modelling are warranted to test more complex models of hypothetical causal pathways.

This study recruited women living in disadvantaged neighbourhoods, a population typically considered ‘hard’ to reach. [29] Further strengths included the longitudinal design and the use of cross-sectional and longitudinal analyses to test theory-based hypothesis. A limitation is the high attrition rate; however, those who withdrew from the study did not differ from those who remained in the study except for their age and region of residence. Further limitations include the use of single mediation models and the relatively long time intervals between the three measurements.

## Conclusion

Results from the cross-sectional analyses provides some evidence that the associations of social support from family and friends and LTPA are mediated by intrapersonal factors, i.e. PA enjoyment, outcome expectations and self-efficacy. However, longitudinal analyses did not confirm this. Future research is needed to determine whether providing or promoting a social supportive network might facilitate LTPA through increases in women’s self-efficacy, PA enjoyment and outcome expectations.

## Supporting information

S1 Table Summary of measures of social support and personal factors. ^1^ For this sample the Cronbach’s alphas were calculated for each of the three measurements (T0-T2)
^2^ Abbreviations: k = Cohen’s kappa coefficient, ICC = intra class correlation coefficient, assessed in an independent sample of 75 women who administered the survey measures twice, a week apart. [25]
^3^ NA = Not applicable, this variable is a sum score of two items
(DOCX)

S2 Table Baseline characteristics of responders and non-responders. *Note*. Responders are those who participated in all three measurements T0, T1 andT2, whereas non-responders are those who only completed the baseline questionnaire (T0). T-test’s and chi-square tests were conducted to compare both groups. Means and standard deviations (sd) or number of participants (N) and proportions (%) are presented for both groups with p-values.
^1^ Low = did not complete high school, Medium = completed high school/trade certificate/diploma, High = completed tertiary education
(DOCX)
