# Supplementary material for: Do intrapersonal factors mediate the association of social support with physical activity in young women living in socioeconomically disadvantaged neighbourhoods? A longitudinal mediation analysis
Source: PLoS One. 2017 Mar 16;12(3):e0173231. doi: 10.1371/journal.pone.0173231 (PMC5354271; doi:10.1371/journal.pone.0173231)
Supplement: S1 Table — 1 For this sample the Cronbach’s alphas were calculated for each of the three measurements (T0-T2) 2 Abbreviations: k = Cohen’s kappa coefficient, ICC = intra class correlation coefficient, assessed in an independent sample of 75 women who administered the survey measures twice, a week apart. [25] 3 NA = Not applicable, this variable is a sum score of two items (DOCX) [file pone.0173231.s001.docx]

## Appendix 1 Summary of measures of social support and personal factors

| *Variable* | *Measurement* | *Scale* | *Internal Consistency ^1^* | *Retest reliability^2^* |
| --- | --- | --- | --- | --- |
| Social support from family | **During the past year, how often did members of your family do PA with you?** | 1 Never  2 Rarely  3 A few times  4 often  5 Very often  6 Not applicable | NA^3^ | ICC = 0.96 |
| Social support from friends | **During the past year, how often did members of your family encourage you to be physically active?** | 1 Never  2 Rarely  3 A few times  4 often  5 Very often | NA^3^ | ICC = 0.84 |
| Enjoyment | **Please rate how you feel at the moment about PA. Below is a list of feelings about PA. :**   - I enjoy it – I hate it - I feel interested – I feel bored - I find it pleasurable – I find it unpleasable - I find it energizing – I find it tiring - It makes me happy – It makes me depressed - I feel good physically while doing it- I feel bad physically while doing it | 1 least enjoyable  7 most enjoyable | α _T1_ = 0.93  α _T2_ = 0.94  α _T3_ = 0.95 | 51-74 % agreement |
| Outcome expectation | **Below are some reasons that you might do regular PA. How important do you think these reasons are for being physically active?:**   - Health - Appearance - Weight - Feeling Fit - Relaxation - Stress relief | 1 No reason at all  2 A slightly important reason  3 A quite important reason  4 A very important reason | α _T1_ = 0.75  α _T2_ = 0.77  α _T3_ = 0.79 | κ= 0.45 – 0.61 |
| Self-efficacy | **How confident are you that you could do PA, in each of the following situations? I’m confident that I could:**   - Do PA even when I’m tired - Do PA even when I’m in a bad mood - Do PA even when I feel I don’t have time - Do PA even when I am on holiday - Do PA even when it is raining | 1 Not at all confident  2 Slightly confident  3 Moderate Confident  4 Very Confident  5 Extremely confident | α _T1_ = 0.80  α _T2_ = 0.83  α _T3_ = 0.86 | κ= 0.33 – 0.49 |

^1^ For this sample the Cronbach’s alphas were calculated for each of the three measurements (T1-T3)

^2^ Abbreviations: k= Cohen’s kappa coefficient, ICC= intra class correlation coefficient, assessed in an independent sample of 75 women who administered the survey measures twice, a week apart. ^16^

^3^ NA = Not applicable, this variable is a sum score of two items
