# Supplementary material for: Do intrapersonal factors mediate the association of social support with physical activity in young women living in socioeconomically disadvantaged neighbourhoods? A longitudinal mediation analysis
Source: PLoS One. 2017 Mar 16;12(3):e0173231. doi: 10.1371/journal.pone.0173231 (PMC5354271; doi:10.1371/journal.pone.0173231)
Supplement: S2 Table — Note. Responders are those who participated in all three measurements T0, T1 andT2, whereas non-responders are those who only completed the baseline questionnaire (T0). T-test’s and chi-square tests were conducted to compare both groups. Means and standard deviations (sd) or number of participants (N) and proportions (%) are presented for both groups with p-values. 1 Low = did not complete high school, Medium = completed high school/trade certificate/diploma, High = completed tertiary education (DOCX) [file pone.0173231.s002.docx]

## Appendix 2 Baseline characteristics of responders and non-responders

| Predictor at baseline (T1) | Responders (N= 271) | Non-responders (N= 926) | *p-*value |
| --- | --- | --- | --- |
| Age [years] (mean ± sd) | 24.7 ± 3.7 | 24.0 ± 3.3 | **0.005** |
| Education level^1^ (N, (%)) |  |  | 0.059 |
| Low | 16 (5.9 %) | 93 (10.3%) |  |
| Medium | 171 (63.3%) | 574 (63.4%) |  |
| High | 83 (30.7%) | 239 (26.4%) |  |
| Marital status (N, (%)) |  |  | 0.262 |
| Married/living as married | 115 (42.4%) | 341 (36.9%) |  |
| Separated/divorced/widowed | 7 (2.6%) | 26 (2.8%) |  |
| Never married | 149 (55.0%) | 556 (60.2%) |  |
| Children living with you (N, (%)) |  |  | 0.147 |
| Yes | 97 (36.2%) | 296 (31.5%) |  |
| No | 171 (63.8%) | 641 (68.5%) |  |
| Area of residence (N, (%)) |  |  | **0.002** |
| Urban | 130 (48.0%) | 544 (58.8%) |  |
| Rural | 141 (52.0%) | 382 (41.3%) |  |
| Leisure-time physical activity (mean ± sd) | 223.1 ± 244.3 | 234.2 ± 300.1 | 0.837 |
| Social support family (mean ± sd) | 6.1 ± 2.2 | 6.0 ± 2.3 | 0.553 |
| Social support friends (mean ± sd) | 5.0 ± 2.3 | 4.8 ± 2.2 | 0.154 |
| Enjoyment for physical activity (mean ± sd) | 31.8 ± 7.3 | 31.2 ± 7.4 | 0.223 |
| Outcome expectations (mean ± sd) | 18.4 ± 3.2 | 18.3 ± 3.4 | 0.900 |
| Self-efficacy (mean ± sd) | 13.6 ± 4.2 | 13.5 ± 4.4 | 0.709 |

*Note.* Responders are those who participated in all three measurements T1, T2 andT3, whereas non-responders are those who only completed the baseline questionnaire (T1). T-test’s and chi-square tests were conducted to compare both groups. Means and standard deviations (sd) or number of participants (N) and proportions (%) are presented for both groups with p-values.

^1^ Low= did not complete high school, Medium= completed high school/trade certificate/diploma, High= completed tertiary education
